# Hypothesis and data-driven dietary patterns and colorectal Cancer survival: findings from Newfoundland and Labrador colorectal Cancer cohort

**DOI:** 10.1186/s12937-018-0362-x

**Published:** 2018-05-25

**Authors:** Ishor Sharma, Barbara Roebothan, Yun Zhu, Jennifer Woodrow, Patrick S. Parfrey, John R. Mclaughlin, Peter Peizhong Wang

**Affiliations:** 1 Division of Community Health and Humanities, Faculty of Medicine, Memorial University of Newfoundland, St. John’s, NF Canada; 2 Clinical Epidemiology Unit, Faculty of Medicine, Memorial University of Newfoundland, St. John’s, NF Canada; 3 Public Health Ontario, Toronto, ON Canada

**Keywords:** Colorectal Cancer, Dietary patterns, Factor analysis, Cluster analysis, Index analysis

## Abstract

**Background:**

Dietary patterns are commonly used in epidemiological research, yet there have been few studies assessing if and how research results may vary across dietary patterns. This study aimed to estimate the risk of mortality/recurrence/metastasis using different dietary patterns and comparison amongst the patterns.

**Methods:**

Dietary patterns were identified by Cluster Analysis (CA), Principal Component Analysis (PCA), Alternate Mediterranean Diet score (altMED), Recommended Food Score (RFS) and Dietary Inflammatory Index (DII) scores using a 169-item food frequency questionnaire.

Five hundred thirty-two colorectal cancer patients diagnosed between 1999 and 2003 in Newfoundland were followed-up until 2010. Overall Mortality (OM) and combined Mortality, Recurrence or Metastasis (cMRM) were identified. Comparisons were made with adjusted Cox proportional Hazards Ratios (HRs), correlation coefficients and the distributions of individuals in defined clusters by quartiles of factor and index scores.

**Results:**

One hundred and seventy cases died from all causes and 29 had a cancer recurrence/metastasis during follow-up. Processed meats as classified by PCA (HR 1.82; 95% confidence interval (CI) 1.07–3.09), clusters characterized by meat and dairy products (HR 2.19; 95% CI 1.03–4.67) and total grains, sugar, soft drinks (HR 1.95; 95% CI 1.13–3.37) were associated with a higher risk of cMRM. Poor adherence to AltMED increased the risk of all-cause OM (HR 1.62; 95% CI 1.04–2.56). Prudent vegetable, high sugar pattern, RFS and DII had no significant association with both OM and cMRM.

**Conclusion:**

Estimation of OM and cMRM varied across dietary patterns which is attributed to the differences in the foundation of each pattern.

**Electronic supplementary material:**

The online version of this article (10.1186/s12937-018-0362-x) contains supplementary material, which is available to authorized users.

## Background

Diet and behavioural factors have crucial roles in the risk and progression of several chronic diseases including colorectal cancer (CRC) [1]. Epidemiological studies on the role of a single nutrient or food items on disease outcome are often inconclusive, which may be in part due to dietary interactions, multi-collinearity [2, 3] and/or inability to detect small effects [4]. Dietary patterns are advantageous in nutritional epidemiology to explore the combined effects of total diet on health and to some extent, overcome these limitations [5]. Dietary patterns not only represent total diet or key factors of diet [6] and the frequency by which foods are habitually consumed, but also reflect an individual’s food preferences modulated by the combination of genetic, cultural, social, health, environmental, behavioural and economic determinants [7].

Data-driven and hypothesis-driven are two major approaches to identify dietary patterns [8]. Cluster and factor analysis are outcome independent empirical data-driven techniques used to determine dietary behaviour in the study population, while index/score-based are hypothesis-driven based on adherence to prior recommendations or guidelines [9].

Briefly, cluster analysis (CA) divides individuals into mutually exclusive, non-overlapping groups based on mean dietary intakes (gm) [10]. Food intake common to all contributes less to cluster formation. Optimal clusters are formed by the maximum ratio of variance across the cluster to within the cluster. No gradient is formed hence comparison is done with the reference cluster. Factorial analysis, specifically Principal Component Analysis (PCA), an exploratory approach, reduces a large set of correlated variables to smaller sets of non-correlated variables, which captures the majority of dietary variations within the study population. Linear combinations are created and each individual receives a score called factors [11]. A higher score represents higher adherence to the particular dietary pattern.

Recommended food score (RFS) [12] and alternate Mediterranean diet score (altMED) [13] are commonly used index-based dietary patterns for which scoring is based on the adherence to the US dietary guidelines and the Mediterranean diet, respectively. Dietary Inflammatory Index (DII) differs to other index-based scales as it doesn’t directly measure the adherence to the established dietary guidelines; instead, it categorizes an individual’s diet into pro- and anti-inflammatory diet based on their dietary response to six inflammatory biomarkers [14]. For such indexes, patterns are derived from gradients, which are then compared to reference quartiles.

Dietary patterns are commonly used in epidemiological research. Studies on how outcome estimation may vary across these different patterns are limited and comparing across the patterns are recommended to better understand disease diet association [15]; however, such studies are limited. This study aimed to use different approaches to identify pre-diagnostic dietary patterns and evaluate and compare their association with the CRC outcome (Overall Mortality (OM) and combined Mortality, Metastasis or Recurrence (cMRM)) using the Newfoundland and Labrador Familial Colorectal Cancer cohort.

## Methods

### Study population

This study used data from the Newfoundland Familial Colorectal Cancer Registry (NFCCR). Five hundred and thirty-two pathologically confirmed (ICD-9 codes: 153.0–153.9, 154.0–154.3, and 154.8 or ICD-10 codes: 18.0–18.9, 19.9, and 20.9) CRC patients diagnosed between 1999 and 2003 and residents of Newfoundland and Labrador, aged 20–75 years, were included in the study. A detailed description of the study population is published elsewhere [16]. Briefly, CRC cases were followed from the date of diagnosis until April 30th, 2010. Overall Mortality (OM; the time between the dates of diagnosis to end of follow-up, or the date of death from all causes until the end of follow-up) and combined Mortality, Metastasis or Recurrence (cMRM; the time between the dates of diagnosis to the end of follow-up, or date of death, recurrence, or metastasis, whichever came first) were calculated.

Individuals who were lost to follow up, still alive or who did not have a recurrence or metastasis by the end of the follow-up period were censored at the time of the last contact. We conducted follow-up questionnaires with participants and linked records to death certificates, pathology reports, autopsy records, physicians’ notes, and surgical reports. Additional data were obtained from the Dr. H. Bliss Murphy Cancer Care Foundation [17]; many of the results can be mutually verified.

### Data collection tools

Participants completing the consent were asked to complete validated food frequency questionnaire (FFQ) [18], personal history questionnaire (PHQ) and some further questions pertaining to family history and medical history. Briefly, the PHQ consisted of 74 questions including the history of bowel screening, medical conditions, use of medications, physical activity, intake of alcohol, tobacco use, socio-demographic information, and reproductive factors for females. Similarly, dietary intake data were collected using a 169-item FFQ retrospectively a year before the diagnosis. For each food item, subjects were asked the frequency of food consumption (daily, weekly, monthly and never scales). Nutrient content was calculated using the Canadian Nutrient File, 2005.

MSI (Microsatellite instability) and BRAF have been associated with cancer prognosis and survival [19, 20]. P V600E BRAF mutation and MSI for the tumour DNA have been determined in a previous study using standard protocol [21]. MSI status was defined as MS high if 30% or more of marker were unstable and MS-stable/MS-low if less than 30% showed instability [22].

### Identifying dietary patterns

For CA, 169 food items were classified into 39 different food groups depending on the ways they are taken and nutrient profile. Food groupings are attached in the Additional file 1: Table S1. Clusters were identified by using K-means non-hierarchical method, an iterative technique which groups data into k clusters in such a way as to maximize the R2 (R2 = 1 − W/T), where W is the sum of squared Euclidean distances between each data point and its within-cluster mean, and T is the sum of squared distances between each data point and the overall mean. FASTCLUS procedure in SAS was applied. Clusters with less than five participants were temporarily removed while forming the stable cluster. A detailed description of cluster formation can be found elsewhere [23]. Overall, four stable clusters were identified. Characteristics of clusters are given in Additional file 1: Table S2.

Three patterns were identified using the PCA correlation matrix as the variables were on different scales. Briefly, exploratory principal component factor analysis was conducted using the same 39 predefined food groups. A varimax rotation (orthogonal) was applied to identify uncorrelated food groups. Factor Eigen-value greater than 1.15, the scree plot and proportion of variance explained were used to identify the number of factors. Patterns were labelled based on factor loading ≥0.5. The factor score of each participant was obtained by summing the intake of each food group multiplied by optimal weights and divided into quartiles. A higher factor score represents greater adherence to that particular dietary pattern. Factor loading and explained variances for three major dietary patterns are shown in Additional file 1: Table S3.

The RFS method developed by Kent, et al. [12] is based on fruits, vegetables, whole grains, lean meats or meat alternatives, and low-fat dairy products. Each individual is given 1 point for each recommended food consumed at least weekly. Based on the FFQ, the maximum score is 47. Total RFS score varies with the number of food items in the FFQ [24]. A higher score represents better adherence to RFS. Details are attached in Additional file 1: Table S4.

The altMED score is based on the Mediterranean diet scale [25]; scoring is based on 9 food groups. If the intake (servings/day) of a particular food group is greater than the median, then it is scored one (versus zero). For red and processed meat, reverse scoring is done. For alcohol, if intake is between 5 and 25 g/d, then it is scored as 1 (versus zero). The maximum altMED score is 9 with a higher score representing better adherence to the altMED diet. Details of the food groups are attached in Additional file 1: Table S5.

Detailed descriptions of the DII score are provided elsewhere [14, 26]. Briefly, a total of 29 nutrient parameters were scored based on their inflammatory response to six inflammatory biomarkers; IL-1β, IL-4, IL-6, IL-10, CRP and tumour necrosis factor (TNF-α). These included carbohydrate, protein, total fat, alcohol, onion, tea, tea (Herbal), pepper, β-carotene, Vitamin B-6, Vitamin B-12, caffeine, cholesterol, energy, fibre, folic acid, iron, Monounsaturated Fatty Acid (MUFA), Polyunsaturated Fatty Acid (PUFA), niacin, magnesium, riboflavin, saturated fatty acid, selenium, thiamine, Vitamin-E, Vitamin-D, vitamin C and zinc. Total DII score obtained is divided into quartiles; higher quartiles represents individuals having diets that are more inflammatory.

### Statistical analysis

Adjusted hazards ratios were estimated using Cox proportional hazard analysis using SAS version 9.4 (SAS Institute, Inc. Cary). Comparisons across patterns were made with adjusted HRs, correlation coefficients and distributions of individuals in clusters by quartile of factor and index scores. Potential confounding factors include age; sex; body mass index (BMI) (classified as < 25, 25–29.99, ≥30 kg/m2); physical activity as measured by the Global Physical Activity Questionnaire (GPAQ) [27]; Metabolic equivalent hours/week (METs/Week, calculated and classified as < 10, 10–50, ≥50); and medical history including cholesterol level; triglycerides; family history of CRC; polyps; diabetes; history of screening; smoking (classified: Yes and No; Yes means smoke at least 1 cigarette/day for 3 months or more); alcohol drinking (classified: standard drink/week; not at all, < 15 and ≥ 15); and regular medication including non-steroidal anti-inflammatory drugs (NSAID), stage and grade of cancer, and reported hormone replacement therapy (HRT, females only). Energy adjustment was completed using the residual method wherever applicable.

The basis for assessing potential confounding factors included: existing evidence, biological plausibility, whether the regression coefficient of the primary variable of interest changed by 10% or more after addition of the potentially confounding variable for every covariate entered in the model at *P* < 0.10. Potential confounders were first selected based on the previous studies as well as a literature search. Initially, potential confounders were screened by the univariate test. Those variables that were statistically non-significant in the univariate test but have an important role in the etiology were included in the model selection step as default; those variables included marital status, the location of a tumor, smoking status, physical activity and reported chemotherapy. As there were too many variables, a stepwise procedure was employed in order to include potential confounding variables that have a detectable effect on the association of interest while retaining the above-mentioned variables in the model.

## Results

### Characteristics of the study population

Mean age of participants and mean age at diagnosis was 62.53 ± 9.06 years and 60.42 ± 9.02 years, respectively. A total of 170 cases died from all causes and 29 had a cancer recurrence or metastasis at the end of the follow-up. Mean time between the date of diagnosis to the end of follow up or date of death from all causes (OM) was 6.27±1.98 years and mean time between the date of diagnosis to the end of follow up or the date of death, recurrence, or metastasis (CMRM) (whichever came first) was 5.70±2.38 years). Almost 68% of the participants were censored for OM and 62.6% for cMRM during analysis.

Table 1 presents the characteristics of the study population with the log-rank test. In the univariate test, there is the significant difference in the OM across the age groups, gender, diagnosis stage and microsatellite instability status. The family history of CRC, reported screening status, history of co-morbidity including diabetes, higher blood cholesterol level, a location of a tumour, smoking status, physical activity and reported chemotherapy had no significant association with the survival.

**Table 1:** Characteristics of study participants with their overall survival status (Univariate); Newfoundland and Labrador Familial Colorectal Cancer Cohort (1999–2003)

Characteristic	No. of patients^a^	No. of deaths (%)	P log-rank
Age at diagnosis			
≤ 60	231	62 (26.83)	
> 60	301	108 (35.88)	0.04
Sex			
Female	211	57 (27.02)	
Male	321	113 (35.20)	0.02
Family history			
Yes	16	6 (37.5)	
No	516	164 (31.78)	0.61
Reported screening procedure			
Yes	68	15 (22.05)	
No	464	155 (33.4)	0.08
Stage at diagnosis			
I/II	311	74 (23.79)	
III/IV	221	96 (43.43)	< 0.001
Diabetes History			
Yes	115	42 (36.52)	0.22
No	417	128 (30.69)	
History of High Cholesterol			
Yes	153	44 (28.75)	
No	349	117 (33.52)	0.44
Tumour location			
Colon	349	105 (30.09)	
Rectum	183	65 (35.51)	0.25
Smoke			
No	139	34 (24.44)	0.07
Yes	393	136 (34.60)	
Physical Activity (Met-hrs/week)			
Low (< 10)	175	52 (29.71)	
Median (10–50)	163	56 (34.35)	0.27
High (50+)	193	62 (32.12)	
MSI status			
MSI-L	440	154 (35.0)	
MSI-H	61	6 (9.83)	< 0.002
Reported chemo Therapy			
Yes	108	42 (38.88)	
No	421	128 (30.40)	0.06

*MSI* Microsatellite instability, *MSI-L* Microsatellite instability low, *MSI-H* Microsatellite instability High ^a^Column total varies due to missing values

### Dietary patterns and survival outcome estimation

Table 2 shows the estimated adjusted hazards ratio corresponding to different dietary patterns with 95% confidence interval. Risk of mortality, recurrence and metastasises varied with the dietary pattern. Four different clusters were identified. When compared with the reference cluster characterized by higher intake of fruits, vegetables, whole grains and wine (Cluster I), the cluster characterized by high intake of meat and dairy products (Cluster II) had a higher risk of cMRM (HR 2.19, 95% 1.03–4.67). The cluster characterized by higher intake of refined grains, sugar/soft drinks (Cluster III) had a higher risk of both cMRM (HR 1.95, 95% 1.13–3.37) and OM (HR 2.05, 95% 1.18–3.57) outcomes. The cluster characterized by the many food groups (Cluster IV) had no significant relation with both OM and cMRM; this cluster was based on many foods as no specific distinguishing or dominating food item could be identified.

**Table 2:** Dietary patterns and Colorectal Cancer Survival (Multivariable adjusted analysis); Newfoundland and Labrador Familial Colorectal Cancer Cohort (1999–2003)

Dietary Pattern Identified by	Combined Mortality, Recurrence and Metastasis		Overall Mortality	
	HR^a^ (95% CI)	P-trend	HR (95% CI)	p-trend
Factor Analysis				
Processed Meat pattern^a^	1.82 (1.07–3.09)*	0.09	1.53 (0.85–2.27)	0.25
Prudent Vegetable Pattern^a^	1.12 (0.69–1.84)	0.62	1.03 (0.61–1.75)	0.90
High-sugar Pattern^a^	1.02 (0.62–1.69)	0.89	1.27 (0.72–2.23)	0.62
Cluster Analysis				
Fruit and Veg, Whole Grain, Fish, wine (Cluster I)	1^c^		1^c^	
Meat, dairy products(Cluster II),	2.19 (1.03–4.67)*		2.04 (0.96–4.35)	
Refined grains, sugar soft Drinks (Cluster III)	1.95 (1.13–3.37)*		2.05 (1.18–3.57)*	
Many foods (Cluster IV)	1.55 (0.92–2.61)		1.50 (0.9–2.56)	
Index Based				
DII^a^	0.89 (0.56–1.42)	0.46	0.78 (0.47–1.25)	0.33
Alt. Mediterranean Diet Score^b^	1.44 (0.92–2.25)	0.08	1.62 (1.04–2.56)*	0.03
Recommended Food Score^b^	1.51(0.92–2.48)	0.06	1.54 (0.92–2.56)	0.045

^a^Hazards Ratio while comparing with highest quartile to the lowest (reference quartile)

^b^Hazards Ratio while comparing with lowest quartile to highest (reference quartile)

^c^Reference group

*Statistically significant

HR ratios Adjusted for energy, stage of cancer, sex, age, marital status, tumour location, screening history, intake of alcohol, radiation and chemo therapy status, Microsatellite instability status wherever applicable

Events are defined as all-cause deaths for overall Mortality and death/recurrence/metastasis (which occurred earliest) for cMRM

Three dietary patterns were identified using PCA: processed meat pattern, prudent vegetable pattern and high sugar pattern. Though the overall trend was not significant (*p* = 0.09), the highest quartile of processed meat pattern significantly increases the risk of cMRM (HR 1.82, 95% CI 1.07–3.09), however, there was no significant association with OM. Neither the prudent vegetable pattern nor the high sugar pattern showed a significant association with both cMRM and OM.

While using index-based patterns, DII and RFS showed no significant association with either OM or cMRM outcomes. The lowest quartile of the altMED score was significantly associated with the higher risk of OM (HR 1.62, 95% 1.04–2.56) but had no significant association with the cMRM.

### Comparison amongst the dietary patterns

Spearman’s correlation coefficients amongst the index-based scores are described in Table 3. Correlations were high and significant because of the similarity in the food items in scoring. A significant positive correlation was observed between RFS and the altMED score (0.60; *p* = 0.001). Significant negative correlations were found between the DII score and the altMED (− 0.601; *p* = 0.001) and RFS (− 0.602; *p* = 0.001) scores.

**Table 3:** Spearman’s Correlation coefficients amongst the index-based score obtained from FFQ; Newfoundland and Labrador Familial Colorectal Cancer Cohort (1999–2003)

	RFS	DII	AltMED Score
RFS	1	−0.61^a^	0.60^a^
		< 0.001	< 0.001
DII		1	−0.61^a^
			< 0.001
AltMED			1

*DII* Dietary inflammatory Index, *AltMed Diet* Alternate Mediterranean diet, *RFS* Recommended Food Score ^a^Significant at 0.05

Table 4 examines the percentage of individuals in the highest quartile of factor and index score in different clusters describing some level of similarity in the foundation of scale. Almost 92% of individuals from the processed meat pattern were in Cluster II characterized by meat and dairy products. Approximately 59% of individuals from the highest quartile of the prudent vegetable pattern were in Cluster I characterized by fruits and vegetables, whole grain, fish and wine. Around 35% of individuals in the highest quartile of high sugar pattern were in the many foods group. In all three index-based patterns, the lowest quartile of DII and highest quartile of altMED and RFS showed the higher proportion of individuals from Cluster I characterized by fruits and vegetables, whole grain, fish and wine (64.97, 36.31 and 57.32% respectively).

**Table 4:** Percentage of individuals in each cluster in highest/lowest quartile of factor/index score; Newfoundland and Labrador Familial Colorectal Cancer Cohort (1999–2003)

	Cluster I	Cluster II	Cluster III	Cluster IV
Characteristics of Cluster	Fruits and Veg, whole grain, fish, wine	Meat, Dairy products, mixed dishes	Sugar/ drinks, total cereals and grains (refined included)	Many foods
Principle Component Analysis^a^				
Processed Meat pattern	15.29	91.89	38.38	16.21
Prudent vegetable pattern	58.60	24.32	6.06	11.46
High Sugar pattern	17.20	10.81	16.16	35.18
Index based				
DII^b^	64.97	13.51	10.1	7.91
AltMed Diet^a^	36.31	8.11	6.06	9.88
RFS^a^	57.32	35.14	13.13	11.86

*DII* Dietary inflammatory Index, *AltMed Diet* Alternate Mediterranean diet, *RFS* Recommended Food Score ^a^Highest Quartile (reference) ^b^Lowest Quartile (reference)

## Discussion

This study is a prospective analysis of mortality among CRC patients diagnosed between 1999 and 2003 from a Canadian population. Both data-driven and hypothesis-driven dietary patterns were determined and relation with CRC patient’s mortality, recurrence and metastasises was estimated. The hypothesis-driven pattern showed how study population is adherent to dietary recommendation while data-driven pattern explains how whole population dietary practice can be classified into different categories. As each dietary pattern was designed to answer the different question, the discrepancy in the outcome estimation was expected despite some level of similarity in the foundation of dietary patterns.

In the current study as identified by CA, the meat and dairy product cluster was associated with increased risk of cMRM while the refined grains, sugar, soft drinks cluster was associated with increased risk of both cMRM and OM. A processed meat pattern as identified by PCA was associated with an increased risk of cMRM. Low adherence to the Mediterranean diet was associated with increased OM. RFS and DII had no significant association with the survival outcomes. The magnitude of estimated HR also varied accordingly.

Epidemiological studies reveal inconsistent results while assessing the relation between dietary patterns and disease outcome in the same population, which is in line with the current study. The study by Reedy J; et al. [15] showed that among male dietary patterns and clusters characterized by fruits, vegetables, lower fats foods, adherence to RFS and MED diet were associated with reduced risk of CRC. Among females, results were inconsistent: meat and potatoes pattern was associated with increased risk and neither MED nor RFS had a significant association.

In the Nurse’s Health study [28] index-based score, AHEI (Alternate Healthy Eating Index) was associated with the lower levels of free oestradiol while no association was found with the patterns identified by factor analysis. In the Health Professionals Follow-up Study cohort, the risk of incident fatal or nonfatal myocardial infarction and stroke (CVD) in the highest quintile of the HEI, alternate HEI, and RFS, respectively, were 28, 39, and 23% lower [29] than the reference quartile, while the highest quintile of a prudent diet score from factor analysis was 30% [30]. While estimating the survival outcome using different dietary pattern, a prospective Danish observational study [31] showed that a prudent diet pattern obtained by PCA was associated with reduced mortality but index-based patterns had no significant association. In the SENECA study, the index based scales including Mediterranean Diet Score (MDS), the Mediterranean Adequacy Index (MDI) and the Healthy Diet Indicator (HDI) showed an inverse association with all-cause mortality [32].

The current study suggested good evidence of comparability between PCA and CA in identifying the dietary pattern as seen in other studies [33, 34] despite their different approach. Almost two-thirds of individuals in the fruit and vegetable cluster (Cluster I) were from the highest quartile of the prudent vegetable pattern identified by PCA having highest loading (> 0.50) for fruits, vegetables, greens, tomatoes and minimal loadings (< 0.15) for processed meat, red meat and refined foods. More than 90% of individuals in Cluster II, characterized by meat and dairy products, were from the highest quartile of the processed meat pattern identified by PCA having higher loading (> 0.5) for red meat, cured processed meat. Similar was the case with other clusters. Despite good evidence of comparability, they are not defined by the same foods, which is likely to be the reason for differential disease outcome estimates.

The hypothesis-driven dietary patterns give higher weight for fruits and vegetables, which is evident by having the majority of individuals in the lowest quartile of DII and the highest quartile of altMED and RFS in Cluster I, characterized by fruits and vegetables. Correlations between index scores were relatively strong and statistically significant as scores were based on similar food recommendations. An increasing score of altMED, RFS and a decrease in DII score are characterized by the higher amount of plant-based food [35].

Indexing systems vary in the definition of optimal diet quality and in their scoring which leads difference in their sensitivity to estimate the disease outcome. Differential classification of food leads to differential exposure. RFS accounts for intake of vegetables, fruits, healthy protein sources, grains and dairy products but does not differentiate between different types of fatty acids or penalize for consumption of foods that are not recommended. Alcohol, energy dense food items and meat products are associated with survival outcomes as seen from empirical approaches but are not considered in scoring. Hence, RFS is likely to underestimate the true association. Further, in the RFS approach, energy cannot be adjusted so the effect of body size, physical activity and higher basal metabolic rate cannot be taken into account for the analysis [36]. Energy adjustment may also help to reduce measurement error [37]. AltMED scoring is based on high consumption of fruits, vegetables, non-refined bread and cereals, legumes and nuts, and moderate consumption of fish, poultry and alcohol. High intake of red and processed meat and saturated fat is penalized during scoring [25]. DII score is based on the inflammatory potential of nutrient/food items in response to the six inflammatory biomarkers. DII is relevant among those diseases associated with chronic inflammation [14]. DII is not only limited to micro and macronutrients but also incorporates commonly used bioactive compounds including flavonoids, spices and tea. Since the current study was based on pre-diagnostic dietary pattern, dietary-induced inflammation may not have a significant role in the risk of mortality.

Multiple reasons could be suggested for the inconsistent results. First, several studies have suggested dietary guidelines have been more strongly related to coronary heart disease than to cancer mortality, even though guidelines are directed toward lowering cancer risk [38]. More extensive studies are done on diet-cardiovascular disease than cancer, and the role of dietary components in cancer causation is still unclear in many instances [29]. Second, dietary guidelines are more effective for cancer incidence than the survival (and therefore mortality) due to the other clinic-pathological factors in determining the cancer survival [38]. Third, the inconsistency might also be due to missing some important components, and some components in the scales may not have a significant association with the cancer risk and survival [29]. Also, our approximation of the three scales varied slightly than the original scale. Original RFS had 23 items and was developed for the all-cause mortality rather than cancer-specific mortality [12]. Likewise, altMED score was developed to assess the variations in the biomarkers level [39] and DII index was based on 45 different food parameters whilst the current study had only 28 parameters [14].

Each method has its own strengths and limitations [6]. Empirical methods are an initial approach and identify dietary patterns as they exist in the population [40] and form the basis for index-based patterns, but suffer certain limitations: (a) They are based on eating behaviour rather than the biological plausibility hence the diet pattern may not exactly reflect disease causation theory [40]; (b) Even though an association is detected, it may not represent beneficial or detrimental eating patterns [41]; (c) Lacks limited reproducibility across the studies [4]; and (d) Includes several arbitrary decisions including consolidation of food items into food groups, number of factors/clusters, method of rotations and labelling of the patterns/clusters [42]. Index-based patterns are based on adherence to the recommendation or guidelines and the foundation of each scale varies. Index-based patterns are generally considered better at estimating the disease outcome as compared to empirical patterns due to their inclusion of relevant and evidence-based components [43]. Results tend to be reproducible across studies. They are limited, however, in that they do not capture a full range of diets in scoring [40] and are difficult to use when scores do not vary considerably within the population [6] and results vary with the cut-offs defined.

### Strengths and limitations of study

This is a prospective follow-up study. Detailed data for the variables (age; sex; marital status; Body Mass Index; screening history; use of medications; co-morbidity status; history of CRC; smoking; physical activity; dietary patterns; alcohol intake; stage and location of tumor; chemotherapy status; etc.) are available including the genetic data on MSI status. Multiple dietary patterns are used for comparison.

This study has a number of limitations. First, the sample is relatively small, which may not offer desirable statistical power and precision in multivariate analysis. Further, cases were followed until 2010 only. Recall error and possible bias are likely to exist as the cases were asked to remember their dietary patterns a year prior to their diagnosis. However, we believe the recall bias, if any, could be non-differential, which is likely to attenuate the observed association. Although bias may exist and sample size is less, it may have little impact on cross-comparison, which is the primary focus of the study. Some cases might have changed their dietary patterns, lifestyle and behaviour after diagnosis or even in the disease induction/latent period; this may lead to possible reverse causation bias, which should be explored in future studies.

## Conclusions

The present study showed that the estimation of OM and cMRM amongst the CRC patients varied with the type of diet pattern used. Hazards ratios for cMRM varied from 1.82; 95% (CI- 1.07-3.09) for processed meat pattern identified by PCA to HR 2.19; 95% CI 1.03–4.67 for cluster characterized by meat and dairy products and HR 1.95; 95% CI 1.13–3.37 for cluster characterized by refined grains, sugar, soft drinks. Only cluster characterized by refined grains, sugar, soft drinks had higher risk of OM (HR 2.05; 95% CI 1.18–3.57). All the diet indices showed similar null associations with both cMRM and OM except Poor adherence to altMED increased the risk of all-cause OM (HR 1.62; 95% CI 1.04–2.56). On the average estimates were higher for data driven methods than hypothesis driven. The variations in the estimated hazards ratios is attributed to the foundation of each dietary pattern identified by various approaches.

## Additional file

Additional file 1: **Table S1.** Food groupings. **Table S2.** Characteristics of Cluster. **Table S3.**. Factor loading and explained variances (VAR) for the three major dietary patterns identified from food frequency questionnaire at baseline using the principal component factor analysis, Newfoundland. **Table S4.** Recommended food Score. **Table S5.** Alternate Mediterranean Diet Score. (DOCX 23 kb)

## Abbreviations

altMED: Alternative Mediterranean diet
BMI: Body mass index
CA: Cluster analysis
CI: Confidence interval
cMRM: Combined mortality, recurrence and metastasis
CRC: Colorectal cancer
DII: Dietary inflammatory index
FFQ: Food frequency questionnaire
FHQ: Family history questionnaire
HRT: Hormone replacement therapy
MUFA: Monounsaturated fatty acid
NFCCR: Newfoundland familial colorectal cancer registry
NL: Newfoundland and Labrador
NSAID: Non-steroidal anti-inflammatory drug
OM: Overall mortality
OR: Odds ratios
PCA: Principal Component analysis
PHQ: Personal history questionnaire
PUFA: Polyunsaturated fatty acid
RFS: Recommended food score
