# Supplementary material for: Hypothesis and data-driven dietary patterns and colorectal Cancer survival: findings from Newfoundland and Labrador colorectal Cancer cohort
Source: Nutr J. 2018 May 25;17:55. doi: 10.1186/s12937-018-0362-x (PMC5968482; doi:10.1186/s12937-018-0362-x)
Supplement: Supplementary file 1 — Table S1. Food groupings. Table S2. Characteristics of Cluster. Table S3.. Factor loading and explained variances (VAR) for the three major dietary patterns identified from food frequency questionnaire at baseline using the principal component factor analysis, Newfoundland. Table S4. Recommended food Score. Table S5. Alternate Mediterranean Diet Score. (DOCX 23 kb) [file 12937_2018_362_MOESM1_ESM.docx]

**Supplementary material**

Supplementary Table S1: Food groupings

| **FG** | **Food Groups** | **Items** |
| --- | --- | --- |
| FG1 | Milk | Whole Milk, 2% Milk, 2% Evaporated Milk, 1% or Skim Milk, Milk Shake |
| FG2 | Yogurt | Yogurt Drink, Yogurt (Plain, 2.4% Fat or More), Yogurt Light (Plain, Less than 2.4% Fat), Yogurt (Fruit Flavoured, Frozen, 2.4% Fat or More), Yogurt Light ( Fruit Flavoured, Frozen, Less than 2.4% |
| FG3 | Coffee | Coffee, Coffee (Decaffeinated), |
| FG4 | Tea | Tea, Tea (Herbal) |
| FG5 | Sugar | Sugar in Tea and Coffee |
| FG6 | Soft drinks | Coca Cola, Pepsi, Other Cola, Diet Soft Drinks, Other Soft Drinks (excluding Diet and Cola) |
| FG7 | Egg | Egg (Boiled), Egg (Fried, Scambled, Omelette) |
| FG8 | Cheese | Cream Cheese, Cheese, Cheese, Light ( 6-15% Fat), Cheese, Ultralight ( 5% or Less), Cottage or Ricotta Cheese |
| FG9 | Mixed dishes | Soups (Creamed), Pasta (with Meat Sauce), Mixed Dishes with Cheese, Pizza (with Meat), Meat Stew, Chili with Meat or Con Carne |
| FG10 | Red Meat | Ground Beef (Regular), Ground Beef (Medium), Ground Beef (Lean), Roast Beef, Steak, Pork Chop, Roast Pork, Baked Ham, Bacon, Veal, Lamb, Hot Dogs or Wieners, Sausage, Corned Beef, Coldcuts, Liver |
| FG11 | Game | Sea-Birds, Seal, Caribou, Moose, Partridge, Other Wild Birds |
| FG12 | Cured/processed red meat | Baked Ham, Bacon, Hot Dogs or Wieners, Sausage, Corned Beef, Coldcuts, Salted/ Dried Meat, Pickled Meat |
| FG13 | Cured/processed meat | Baked Ham, Bacon, Hot Dogs or Wieners, Sausage, Corned Beef, Coldcuts, Fried Chicken, Salted/ Dried Meat, Pickled Meat, Fried Fish, Canned Fish, Smoked Fish or Lox, Salted or Dried Fish, Pickled Fish |
| FG14 | Polutry | Fried Chicken, Chicken/Turkey, Chicken/Turkey, Skin Removed |
| FG15 | Fish | Shellfish, Fried Fish, Fish (Baked or Broiled), Canned Fish, Smoked Fish or Lox, Salted or Dried Fish, Pickled Fish |
| FG16 | Processed Fish | Canned Fish, Smoked Fish or Lox, Salted or Dried Fish, Pickled Fish |
| FG17 | Fruit Juice | Orange or Grapefruit Juice, Apple or Grape Juice, Other Fruit Juices (Pineapple, Cranberry, etc), Fruit Drink/ Lemonade, Fruit Drinks/ Iced Tea |
| FG18 | Other Fruit | Apples, Pears, Grapes, Bananas, Peaches, Plums, Nectarine, Apricot, Canteloupe, Watermelon, Honeydew Melon, Mango, Papaya, Applesauce, All other Fruit |
| FG19 | Root Vegetables | Potatoes, French Fries and Fried Potatoes, Carrots, Turnips or Rutabagas, Other Root Vegetables |
| FG20 | Cruiferous vegetables | Broccoli, Cabbage, Coleslaw, Cauliflower, Asparagus or Brussel Sprouts |
| FG21 | Other Greens | Spinach / other Green Leafy Vegetables, Green Salad (with Lettuce) |
| FG22 | Beans, peas | Peas or Lima Beans, Green Beans, Beans or Lentils, Pea Soup |
| FG23 | Tomato Sauce | Tomatoes (Fresh), Tomatoes (Canned, Pureed, Sauce), Ketchup |
| FG24 | Other Vegetables | Corn, Cucumber, Onions (Raw or Cooked), Beets (Boiled or Pickled)  Yellow Squash, Zucchini or eggplant, Sweet Pepper, Bean Sprouts, Alfalfa Sprouts, Avocado, Other Vegetables (Celery, Mushrooms |
| FG25 | Total Cereals and Grains | Bran or Granola Cereals, Whole Wheat Cereals, Cereals, Not Sugar Coated, Hot Cereals, Sugar Coated Cereals, Other Breakfast Cereals, Sugar on Cereal, 100% Whole Grain or Dark Bread, 60% Whole Grain, Light Rye, White Bread, White Bread Rolls (Including Hot Dog Buns), Whole Wheat Rolls, Crackers, Bran / Oat Muffin, Other Muffin, Pancakes, Waffles, Macaroni, Spaghetti, Noodles, etc, Rice, Crisp Snacks |
| FG26 | Whole grains | Whole Wheat Cereals, 100% Whole Grain or Dark Bread, 60% Whole Grain, Light Rye, Whole Wheat Rolls |
| FG27 | Desserts and sweets | Cakes, Pies and Tarts, Donuts and Sweet Rolls, Cookies, Ice Cream  Light or Diet Ice Cream, Pudding, Diet or Light Pudding, JELLO, Popsicles, Freezies, Chocolate Bar and Candy, Candy (without Chocolate) |
| FG28 | Vegetable Juice | Vegetable Juices |
| FG29 | Beer | Beer or Ale |
| FG30 | White wine | White Wine |
| FG31 | Red wine | Red Wine, Sherry, Port(or Other Fortified Wine) |
| FG32 | Liquor | Liquor |
| FG33 | Citrus | Citrus Fruits |
| FG34 | Berries | Berries |
| FG35 | Dried fruit | Dried Fruits |
| FG36 | Canned Fruit | Canned Fruit |
| FG37 | Pies, tarts | Pies and Tarts |
| FG38 | Jam, Jelly | Jam, Jelly, Honey, Syrup |
| FG39 | Pickled Vegetables | Pickles, Relish |

Supplementary Table S2: Characteristics of Cluster

| **Cluster I (157)** | **Cluster II (37)** | **Cluster III(99)** | **Cluster IV (253)** |
| --- | --- | --- | --- |
| Fruits | Dairy products  (Milk, cheese, Yogurt) | Desserts and sweets | Did not indicate any specific distinguishing food as it had no specific dominating food items. |
| Fruit and Vegetable Juice | Red and Processed meats/fish, game meat | Sugar |  |
| Vegetables, greens | Poultry | Soft Drinks |  |
| Beans, peas | Mixed Dishes | Total grains and cereals |  |
| Whole Grains |  |  |  |
| Wine (Red, white) |  |  |  |
| Fish (Non-processed) |  |  |  |
| *Numbers in the parenthesis indicates the cluster size* | | | |

Supplementary Table S3: Factor loading and explained variances (VAR) for the three major dietary patterns identified from food frequency questionnaire at baseline using the principal component factor analysis, Newfoundland

| **Food Groups** | **Processed Meat pattern** | **Prudent Vegetable pattern** | **High-Sugar Pattern** |
| --- | --- | --- | --- |
| Milk |  | 0.19 |  |
| Yogurt |  | 0.31 |  |
| Sugar |  | -0.19 | 0.20 |
| Tea |  |  | 0.17 |
| Coffee | 0.17 |  |  |
| Soft Drinks | 0.19 |  |  |
| Cheese | 0.15 | 0.21 |  |
| Egg | 0.21 |  | 0.16 |
| Mixed Dishes | 0.31 | 0.17 | 0.23 |
| Red Meat | **0.69** |  | 0.17 |
| Cured/processed red meat | **0.73** |  | 0.21 |
| Cured/processed meat | **0.93** |  |  |
| Game | 0.23 |  |  |
| Poultry | 0.22 | 0.27 |  |
| Fish | **0.58** | 0.32 | -0.22 |
| Processed Fish | **0.50** | 0.25 |  |
| Fruit Juice |  | 0.24 | 0.23 |
| Root Vegetables | 0.28 |  | 0.15 |
| Cruciferous vegetables |  | **0.54** |  |
| Other Fruits |  | **0.59** |  |
| Other greens |  | **0.60** | -0.22 |
| Tomato Sauce |  | **0.50** |  |
| Other Vegetables | 0.22 | **0.54** |  |
| Beans, Peas | 0.15 | 0.25 |  |
| Pickled Vegetables | 0.15 | 0.26 | 0.15 |
| Total Cereals and Grains | 0.23 | 0.38 | 0.28 |
| Whole grains |  | 0.33 |  |
| Citrus |  | 0.34 |  |
| Berries |  | 0.45 |  |
| Dried Fruits |  | 0.39 |  |
| Vegetable Juice |  | 0.17 |  |
| Beer | 0.19 |  |  |
| White Wine |  |  |  |
| Red Wine |  |  |  |
| Liquor |  |  |  |
| Desserts and Sweets | 0.31 |  | **0.63** |
| Pies, Tarts | 0.15 |  | **0.54** |
| Canned Fruits |  | 0.21 | 0.23 |
| Jam, Jelly |  |  | 0.26 |
| Proportion o f VAR explained (%) | 37.79 | 22.93 | 11.10 |
| Cumulative VAR Explained (%) | 37.79 | 62.72 | 73.82 |

Absolute loadings < 0.15 were not listed for simplicity. Those with loadings of 0.50 or greater are in bold

Supplementary Table S4: Recommended food Score

| Food Group | Foods Included |
| --- | --- |
| Vegetables | Potato, Carrot, Broccoli, Cabbage, Cauliflower, Corn, Peas/lime beans, Green beans, Beans/lentils, Spinach, Green Salad, Cucumber, Tomato, Canned Tomato, Onions, Beets, Turnip/rutabagas, Root Vegetables, Yellow Squash, Zucchini/egg plant, Avocado, Veg Juice |
| Fruit | Apple/pears, citrus fruits, Berries, Grapes, Bananas, (Peaches, Plums, Apricots), Dried Fruits, Apple Sauce, Cantaloupe, Watermelon, Honeydew melon, mango, papaya, fruit juice, |
| Protein | Chicken/turkey without skin, Shell fish, other fish, canned tuna, tofu |
| Grains | Bran, whole wheat, hot cereal, cereals, dark bread, whole grains |
| Dairy | Skim milk |
| Maximum Score | 47 *(1 point for each item consumed at least weekly)* |

Supplementary Table S5: Alternate Mediterranean Diet Score

| Vegetables | All Vegetables except Potato | Greater than median intake (g/d) |
| --- | --- | --- |
| Legumes | Tofu, beans, peas, lentils | Greater than median intake (g/d) |
| Fruit | All fruit and Juices | Greater than median intake (g/d) |
| Nuts | Nuts | Greater than median intake (g/d) |
| Whole Grains | Bran, whole wheat, cereals, hot cereals, dark bread, whole grain | Greater than median intake (g/d) |
| Red and Processed Meat | Red and Processed Meat | Less than median intake (g/d) |
| Fish | All fish and shell fish | Greater than median intake (g/d) |
| Ratio Mono-unsaturated to saturated fat |  | Greater than median intake |
| Alcohol |  | 5-25g/d |

*0 point if the criteria is not met
Maximum Score 9*
